# Bactericidal and virucidal activity of ethanol and povidone‐iodine

**DOI:** 10.1002/mbo3.1097

**Published:** 2020-06-22

**Authors:** Andreas Sauerbrei

**Affiliations:** ^1^ Section Experimental Virology Institute of Medical Microbiology Jena University Hospital Jena Germany

**Keywords:** bactericidal/virucidal activity, ethanol, exposure time, literature data, PVP‐I, quantitative suspension test

## Abstract

Ethanol and povidone‐iodine (PVP‐I) are important microbicides that inactivate bacteria and viruses. The present study provides a review of literature data on the concentration‐dependent bactericidal and virucidal activity of ethanol and PVP‐I in vitro. A systematic search was performed using the meta‐database for biomedicine PubMed. Eventually, 74 studies with original data on the reduction of bacterial and viral infectivity using in vitro tests were analyzed. A safe bactericidal effect of ethanol can be expected at concentrations between 60% and 85%, and the exposure times vary between ≤0.5 and ≥5 min. Within an exposure of up to 5 min, 80%–90% ethanol also exerts virucidal/low‐level activity, which includes its action against enveloped viruses plus adeno‐, noro‐, and rotaviruses. For PVP‐I, the best bactericidal and virucidal/high‐level effect is present at a concentration range of approx. 0.08%–0.9% depending on the free iodine concentration. The maximum exposure times are 5 min for bacteria and 60 min for viruses. The available data may help optimize the significant inactivation of bacteria and viruses in various areas. However, as the conditions in application practice can vary, concrete recommendations for the application can only be derived to a limited extent.

## Graphical abstract

The present study provides an overview of the bactericidal and virucidal activity of ethanol and povidone‐iodine based on a systematic literature search. Both ethanol and povidone‐iodine have an inactivating effect on bacteria and viruses depending on the concentrations used. The data may help to improve the microbicidal application of ethanol and povidone‐iodine in practice.

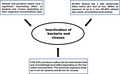

## 1 INTRODUCTION

Ethanol and povidone‐iodine (PVP‐I) are important active components of disinfectants or antiseptic agents, particularly used in the field of medicine and in the public health sector to prevent the spread of infectious agents. Ethanol is widely used as a hand disinfectant, mainly in gels, hand rubs, and foams (Goroncy‐Bermes, Koburger, & Meyer, 2010; Kampf, Marschall, Eggerstedt, & Ostermeyer, 2010; Kramer, Rudolph, Kampf, & Pittet, 2002). The target pathogens of these antiseptic agents include bacteria, yeast, and enveloped viruses (Kampf & Kramer, 2004). The World Health Organization, the US Food and Drug Administration, and the Centers for Disease Control and Prevention consider the use of ethanol at concentrations between 60% and 95% as effective and safe and, therefore, as essential for hand rubbing (Boyce & Pittet, 2002; U.S. Food & Drug Administration, 2019; World Health Organization, 2009). In a limited number of experimental studies, ethanol has thus far been tested for its bactericidal efficacy. It has been shown that 85% ethanol, in particular, demonstrated a comprehensive bactericidal effect within a short time of 15 s (Kampf & Hollingsworth, 2008). In comparison, several studies have tested virucidal efficacy including limited virucidal activity (active against enveloped viruses), a low‐level of virucidal activity (active against enveloped viruses plus adeno‐, noro‐, and rotaviruses), and a high‐level of virucidal activity (active against enveloped and non‐enveloped viruses). In most studies, only a limited virucidal activity was detected for higher ethanol concentrations.

The iodophor PVP‐I, consisting of elementary iodine bound to the carrier poly(1‐vinyl‐2‐pyrrolidone), is regarded as a microbicide that exerts broad‐spectrum activity against bacteria, fungi, protozoa, and viruses (Görtz, Reimer, & Neef, 1996). Due to its excellent antiseptic properties, it is used particularly for wound, skin, and throat disinfection. The number of experimental studies testing different concentrations of PVP‐I from <0.001% to 10% for inactivating efficacy against gram‐positive and gram‐negative bacteria is extensive. Significantly, the inactivating effect is dependent on the concentration of free iodine, which decreases with the increasing concentration of PVP‐I especially within the range of 5%–10% (Atemnkeng, Plaizier‐Vercammen, & Schuermans, 2006). Similarly, an increasing number of studies in the literature are testing the spectrum of virucidal efficacy from limited virucidal activity to a high‐level of virucidal activity.

The objective of the present study was to describe the bactericidal and virucidal activity of ethanol and PVP‐I without the addition of interfering substances (organic load) based on the data available in the literature. Particular attention should be given to an exposure temperature of 22 ± 3°C and an exposure time of up to 60 min.

## 2 MATERIAL AND METHODS

First, a search and analysis of the existing literature were carried out from January to March 2019 using PubMed (the English‐language text‐based meta‐database for biomedicine) with the keywords “bactericidal activity/efficacy of ethanol”, “virucidal activity/efficacy of ethanol”, “bactericidal activity/efficacy of povidone‐iodine”, “virucidal activity/efficacy of povidone‐iodine”. About 600 entries were found under these keywords. All studies with original data on the reduction of bacterial and viral infectivity using in vitro tests were selected. After the analysis of the respective abstracts, 148 publications were shortlisted, and their full text had to be evaluated. In the cited literature of these articles, another 50 relevant papers were found; the full text of these papers was also analyzed. From these 198 papers, 74 publications resulted, which were of essential importance in defining the bactericidal and virucidal activity of ethanol and PVP‐I. To be able to make a statement about the concentration‐dependent antimicrobial effect of ethanol and PVP‐I, their respective concentrations analyzed in the literature were evaluated with respect to their bactericidal and virucidal effect. Only studies that tested the bactericidal or virucidal efficacy of ethanol or PVP‐I in liquids were included. Studies that analyzed disinfectants based on ethanol or PVP‐I, but with additives that may influence the microbicidal effect, were excluded from the present review.

Methodologically, only those studies were considered that had examined the listed results in in vitro tests. A compilation of these methods and the corresponding references are given in Table 1 for the determination of bactericidal efficacy and in Table 2 for the determination of virucidal efficacy. The most frequently used method for determining the bactericidal and virucidal effect of ethanol and PVP‐I was the quantitative suspension test, which was often carried out in the standardized form following European standards or national guidelines. In a few cases, carrier tests of practical relevance using glass or metal carriers or ex vivo skin tests with pigskin were also used.

**TABLE 1:** Methods for evaluation of bactericidal efficacy of ethanol and PVP‐I

Method	References
Quantitative suspension test	Adams, Quayum, Worthington, Lambert, and Elliott (2005); Anagnostopoulos et al. (2018); Atemnkeng et al. (2006); Berkelman et al. (1982); Ghogawala and Furtado (1990); Haley, Marling‐Cason, Smith, Luby, and Mackowiak (1985); Heiner et al. (2010); Kasuga, Ikenova, and Okuda (1997); McLure and Gordon (1992); Musumeki et al. (2018),; Nakagawa et al. (2006); Reybrouck (1985); Sanchez et al. (1988); Shiraishi and Nakagawa (2002); Suzuki et al. (2012); Tavichakorntrakool et al. (2014); Wichelhaus et al. (1998); Wutzler et al. (2000)
Quantitative suspension test, 32°C	Hill and Casewell (2000)
Quantitative suspension test, prEN12054, ethanol w/w	Kampf, Rudolf, Labadie, and Barrett (2002)
Quantitative suspension test, ethanol w/w	Kampf and Hollingsworth (2008)
Quantitative suspension test, ethanol v/v	Kida (2009); Koshiro and Oie (1984)
Quantitative suspension test, EN1276	Messager, Goddard, Dettmar, and Maillard (2001)
Quantitative suspension test, EN1276, with BSA or serum	Møretrø et al. (2009); Rikimaru, Kondo, Kondo, and Oizumi (2000), Rikimaru et al. (2002)
Quantitative suspension test, DGHM 1991	Reimer et al. (2000, 2002)
Quantitative suspension test, EN 13727 with BSA and erythrocytes	Salvatico et al. (2015); Eggers et al. (2018)
Quantitative suspension test, EN1040, EN1275	Smock, Demertzi, Abdolrasouli, Azadian, and Williams (2018)
Microdilution assay	Anderson, Horn, Lin, Parks, and Peterson (2010)
Glass‐carrier test	Messager et al. (2001)
European surface test, EN13697 with BSA, ethanol n.d.	Møretrø et al. (2009)
Carrier test, EN 14561	Schedler et al. (2017)
Colony‐counting method	Shimizu et al. (2002)
Fluorescence microscopy	Wutzler et al. (2000)
Ex vivo skin test	Messager et al. (2001); Nishioka et al. (2018)

Abbreviations: BSA, bovine serum albumin; DGHM, Deutsche Gesellschaft für Hygiene und Mikrobiologie; EN, European Norm; n.d., no data; v/v, volume per volume, vol%; w/w, weight per weight, weight%.

**TABLE 2:** Methods for evaluation of virucidal efficacy of ethanol and PVP‐I

Method	References
Quantitative suspension test	Boudouma, Enjalbert, and Didier (1984); Ito et al. (2006); Iwasawa, Niwano, Kohno, and Ayaki (2012); Kampf et al. (2002); Kawana et al. (1997); Matsuhira et al. (2012); Noda et al. (1981); Pfaender et al. (2015); Wada et al. (2016); Wutzler et al. (2000)
Quantitative suspension test, ethanol n.d.	Belliot, Lavaux, Souihel, Agnello, and Pothier (2008); Wolff, Schmitt, Rahaus, and König (2001)
Quantitative suspension test, ethanol v/v	Duizer et al. (2004); Doultree, Druce, Birch, Bowden, and Marshall (1999); Paulmann et al. (2011)
Quantitative suspension test, 33°C	Yates et al. (2019)
Quantitative suspension test EN 14476, ethanol v/v	Sauerbrei, Eschrich, Brandstädt, and Wutzler (2009); Steinmann, Paulmann, Becker, Bischoff, and Steinmann (2012)
Quantitative suspension test EN 14476, ethanol n.d.	Ciesek et al. (2010)
Quantitative suspension test EN14476 with BSA and erythrocytes	Eggers, Eickmann, Kowalski, Zorn, and Reimer (2015), Eggers, Eickmann, and Zorn (2015)
Quantitative suspension test, testing of ECHO‐11 with serum, ethanol n.d.	Kurtz, Lee, and Parson (1980)
Quantitative suspension test, German DVV/RKI guideline, 1990, ethanol v/v	Gehrke et al. (2004); Sauerbrei et al. (2004); Wutzler, Sauerbrei, Klöcking, Brögmann, and Reimer (2002)
Quantitative suspension test, German DVV/RKI guideline, 2005	Sauerbrei, Schacke, Glück, Egerer, and Wutzler (2006)
Quantitative suspension test, German DVV/RKI guideline, 2008, ethanol v/v	Sauerbrei et al. (2009)
Quantitative suspension test, German DVV/RKI guideline, 2009, ethanol v/v	Rabenau et al. (2010); Sauerbrei et al. (2012); Sauerbrei and Wutzler (2010)
Carrier test, Ethanol n.d.	Doerrbecker et al. (2011); Malik, Meherchandani, and Goyal (2006); Saknimit et al. (1988); Whitehaed and McCue (2010)
Carrier test, Ethanol v/v	Tyler and Ayliffe (1987); Tyler, Ayliffe, and Bradley (1990)
Carrier test with BSA and erythrocytes, ethanol v/v	Eterpi, McDonnell, and Thomas (2009); Magulski et al. (2009)
Ultrafiltration	Boudouma et al. (1984)
Analysis of NoV‐VLPs by transmission electron microscopy, ethanol n.d.	Sato et al. (2016)

Abbreviations: BSA, bovine serum albumin; DVV, Deutsche Vereinigung zur Bekämpfung der Viruskrankheiten; EN, European Norm; n.d., no data; NoV‐VLP, Human Norovirus‐like particles; RKI, Robert Koch‐Institute; v/v, volume per volume, vol%.

In the evaluation of the data obtained, primarily results were considered that were obtained without interfering additives to aggravate the disinfection effect. If such findings were not available, the obtained results were analyzed with the addition of interfering substances (organic load, e.g., bovine serum albumin or erythrocytes), as shown in Tables 1 and 2. The exposure temperature in the studies considered was 22 ± 3°C in most of the cases. In some studies, only the term “room temperature” was used; alternatively, no precise information on the exposure temperature was given, in which case room temperature was assumed. Deviations from the specified temperature range are also noted in Tables 1 and 2.

For the analysis of the microbicidal activity of ethanol and PVP‐I in the listed studies, various initial compounds in the form of commercial disinfectants or antiseptics were used, as noted in the tabular lists of the results on antimicrobial activity using footnotes. Where no note is given, either ethanol or PVP‐I were used as chemical reagents. The concentration of ethanol was given by most investigators in volume percent (vol%, v/v—volume per volume), and, in very rare cases, in weight percent (weight%, w/w—weight per weight). In numerous studies, however, the tested concentrations of ethanol were not specified in greater detail (see Tables 1 and 2, n.d.—no data). The stated concentrations of PVP‐I generally refer to w/v (weight per volume).

The range of activity of disinfectants against enveloped/lipophilic viruses is called “limited virucidal,” and the range of activity against enveloped/lipophilic, as well as non‐enveloped/hydrophilic, viruses is called “virucidal” (Rabenau et al., 2014). As per current German guidelines or recommendations (Rabenau, Schwebke, Steinmann, Eggers, & Rapp, 2012), the “virucidal” range is further subdivided into “virucidal/low‐level” or “limited virucidal plus” (enveloped viruses in addition to adeno‐, noro‐, and rotaviruses, but excluding entero‐ and parvoviruses) and “virucidal/high‐level” (all viruses mentioned as virucidal/low‐level plus entero‐ and parvoviruses). As per the European terminology, there are also three different claims on virucidal activity: “active against enveloped viruses”; “limited spectrum of virucidal activity” including against enveloped viruses plus adeno‐, noro‐, and rotaviruses”; and “virucidal activity,” which includes action against all relevant human viruses (EN, 14476, 2019).

## 3 RESULTS

Table 3 provides a summary overview of the concentration‐dependent inactivating effect of ethanol on bacteria and viruses. Detailed results of the studies analyzed from the literature are shown in Tables A1 and A2. European and American guidelines (Eggers, Koburger‐Janssen, Eickmann, & Zorn, 2018; Heiner, Hile, Demons, & Wedmore, 2010; McLure & Gordon, 1992; Reimer et al., 2000; Salvatico, Feuillolay, Mas, Verrière, & Roques, 2015) generally assume a safe bactericidal effect if the tested substance causes a reduction in the bacterial count by 4–5 powers of ten (4–5 log_10_) corresponding to 99.99%–99.999% (see Tables A1 and A3). In several cases, a reduction in the bacterial count by 3 powers of ten (3 log_10_) corresponding to 99.9% (Anagnostopoulos et al., 2018; Rikimaru et al., 2002) or complete germ inactivation (100%) (Berkelman, Holland, & Anderson, 1982; Kampf & Hollingsworth, 2008; Koshiro & Oie, 1984; Tavichakorntrakool et al., 2014) is also given in the literature. According to the current guidelines, a virucidal effect is defined as a reduction of the virus titer by at least 4 decimal powers (≥4 log_10_) resulting in virus titer reduction of ≥99.99% (Eggers et al., 2018; Kawana et al., 1997; Noda, Watanabe, Yamada, & Fujimoto, 1981; Rabenau, Rapp, & Steinmann, 2010; Sauerbrei et al., 2012; Yates, Shanks, Kowalski, & Romanowski, 2019) (see Tables A2 and A4). Only one study describes a complete (100%) virus inactivation by the electron microscopic analysis of human norovirus‐like particles (Sato et al., 2016). When analyzing the results in relation to the concentrations of the active substance, it must be taken into account that when using the quantitative suspension test to determine the virucidal effect, the final concentration of the formulation tested is usually 80% (EN, 14476, 2019; Rabenau et al., 2014).

**TABLE 3:** Summary of bactericidal and virucidal efficacy of ethanol as a function of substance concentration

Concentration (%)	Spectrum of activity	Yes/no
30	Bactericidal	Not safe even with long exposure time of ≥30 min (limited data)
	Virucidal	No
40–50	Bactericidal	**Probably yes, but longer exposure time of >5 min (few data)**
	Virucidal (limited virucidal)	**Yes: enveloped/lipophilic viruses exposure time ≤5 min**
		No: non‐enveloped/hydrophilic viruses
60–70	Bactericidal	**Yes, longer exposure time of ≥5 min necessary**
	Virucidal (limited virucidal)	**Yes: enveloped/lipophilic viruses exposure time ≤1 min**
		No: non‐enveloped/hydrophilic viruses
80–85/90	Bactericidal	**Yes, optimal concentration, exposure time ≤0.5 min**
	Virucidal (virucidal/low‐level or limited virucidal plus)	**Yes, optimal concentration, exposure time up to 5 min** (partly insufficient for enteroviruses and other non‐enveloped viruses)
100	Bactericidal	No
	Virucidal	No

It is demonstrated in Table 3 that a safe bactericidal effect of ethanol, including inactivation of vegetative forms of spores, is given in concentrations of 60%–85%, with the optimal effective concentration being 80%–85%. In the latter concentration range, exposure times are a maximum of 30 s, and for 60%–70% ethanol, a longer exposure of ≥5 min is necessary. Concentrations of 30%–50% ethanol have a significantly lower bactericidal activity, whereas the tested exposure times of 5–30 min are partly insufficient for a significant bactericidal effect. A concentration of 80%–90% ethanol also exerts virucidal/low‐level activity, which includes action against enveloped viruses plus adeno‐, noro‐, and rotaviruses. For a titer reduction of 4 log_10_, a time interval of up to 5 min is required, depending on the virus structure, whereby a safe virucidal effect against enteroviruses could not be demonstrated. In comparison, lower concentrations of 60%–70% ethanol exert an inactivating effect on enveloped (lipophilic) viruses, whereas non‐enveloped (hydrophilic) viruses are not sufficiently inactivated in this concentration range or are partially inactivated only during long exposure times. Ethanol at 40%–50% inactivates most enveloped viruses within 5‐min exposure. For the inactivating effect of >90% ethanol, there exist inadequate, or no meaningful, data. Concentrations of 100% ethanol do not have any safe bactericidal and virucidal effects.

A summary overview of the concentration‐dependent inactivating effect of PVP‐I at concentrations of ≤0.001%–10% on both bacteria and viruses is provided in Table 4. Detailed results of the studies analyzed from the literature are presented in Tables A3 and A4. For bacteria, as for viruses, a similar concentration‐dependent effect exists. The best bactericidal and virucidal effect of PVP‐I is manifested at a concentration range of approx. 0.08%–0.9%. The maximum exposure times are 5 min for bacteria and 60 min for viruses (poliovirus type 1, adenoviruses), depending on the virus structure. However, for this concentration range, gram‐positive cocci have also been described in the literature, which were not inactivated within 1 min (see Table A3), and exposure times beyond this were not tested. Although lower concentrations of 0.009%–0.05% PVP‐I have a moderate inactivating effect on bacteria and reduced action on viruses, only a few studies are available, which, on average, usually describe longer exposure times as well as ineffectiveness within short exposure times. Concentrations of 1%–5% PVP‐I also exert optimum bactericidal and virucidal activity, although the activity decreases slightly with increasing PVP‐I concentration; additionally, longer exposure times (bacteria up to 30 min, viruses up to 60 min) are necessary. PVP‐I at concentrations of 6%–10% shows moderate microbicidal activity; however, especially at a concentration of 9%–10% PVP‐I, the significant inactivation of gram‐positive cocci and poliovirus type 1 is uncertain. Since various initial compounds were used in the studies considered from the literature for testing PVP‐I, individual concentrations may show slight deviations to their antimicrobial effect. PVP‐I at concentrations of ≤0.001 has no inactivating effect against bacteria and viruses. Concentrations in this range have rarely been tested (data not listed).

**TABLE 4:** Summary of bactericidal and virucidal efficacy of PVP‐I as a function of substance concentration

Concentration (%)	Spectrum of activity	Yes/no
≤0.001	Bactericidal	No
	Virucidal	No
0.009–0.05	Bactericidal	**Yes** →
	Virucidal (virucidal/high‐level)	No
0.08–0.9	Bactericidal	**Yes** ↑↑, maximal exposure time 5 min
	Virucidal (virucidal/high‐level)	**Yes** ↑↑, maximal exposure times 60 min
1.0–5.0	Bactericidal	**Yes** ↑, maximal exposure times 30 min
	Virucidal (virucidal/high‐level)	**Yes** ↑, maximal exposure times 60 min
6.0–10.0	Bactericidal	**Yes** →
	Virucidal (virucidal/high‐level)	**Yes** →

Note

→ moderate activity (partially ineffectiveness), ↑ good activity, ↑↑ very good activity.

## 4 DISCUSSION

The present article aimed to describe the bactericidal and virucidal activity of ethanol and PVP‐I as a function of substance concentration without the addition of organic load at an exposure temperature of 22 ± 3°C in in vitro tests. A safe bactericidal effect of ethanol can be expected at concentrations between 60% and 85%. For 60%–70% ethanol, exposure times of ≥5 min are necessary, while for concentrations of 80%–85% ethanol, a ≤0.5 min exposure is effective. Hence, the latter range can be regarded as the optimal concentration for the bactericidal activity of ethanol. Bactericidal activity of 40%–50% ethanol is probable within exposure times longer than 5 min. However, data on the bactericidal effect at these concentrations are only available from two studies in the literature in which maximum exposure times of 5 min in the quantitative suspension test under protein load were used (Koshiro & Oie, 1984; Møretrø et al., 2009). The reason for the small number of studies is that ethanol is mainly used for hand and skin disinfection with short application times, and, therefore, testing of longer exposure times is usually not necessary. A contact time of 30 s is recommended for hygienic hand disinfection (EN 1500, 2013) and 90 s for surgical hand disinfection (EN12791:2016+A1:2017, 2017). The testing of concentrations <80% ethanol has little practical relevance, as ethanol as a single component is only effective within short exposure times at higher concentrations (Kampf & Hollingsworth, 2008); alternatively, it is effective as low‐concentration ethanol only in combination products, for instance in combination with propanol (Marchetti, Kampf, Finzi, & Salvtorelli, 2003). However, 100% of ethanol has no safe microbicidal effect, as the denaturation of proteins is difficult to achieve in the absence of water (Gold & Avva, 2020). Nevertheless, a study published by Koshiro and Oie (1984) reported the complete inactivation of gram‐negative and gram‐positive bacteria except for *Staphylococcus aureus* by 99.5% ethanol in quantitative suspension tests.

A complete virucidal/high‐level efficacy cannot be achieved with certainty by ethanol at any concentration. The best effect has been reported at concentrations of 80%–90% ethanol. This comprises action against enveloped viruses plus adeno‐, noro‐, and rotaviruses within 5‐min exposure defined as virucidal/low‐level or limited virucidal plus. However, for several non‐enveloped viruses such as enteroviruses, the concentration range is not effective or longer exposure is necessary. The feline calicivirus often used as a surrogate for human noroviruses seems to be inactivated significantly using 80% ethanol (Gehrke, Steinmann, & Goroncy‐Bermes, 2004). For ethanol concentrations >90%, the current data situation is very limited. This is mainly since these concentrations cannot be tested in the quantitative suspension test under current guidelines. This has not been considered in a recent publication on the efficacy of ethanol against viruses in hand disinfection (Kampf, 2018). Lower ethanol concentrations of 60%–70% with ≤5 (10) min exposure exert limited virucidal activity comprising of action against only enveloped, but medically relevant, viruses such as the herpes simplex, influenza A, and hepatitis C viruses (Doerrbecker et al., 2011; Noda et al., 1981). However, literature data are only available for short exposure times of maximum 10 min in suspension and carrier tests with, and without, protein load. Interestingly, 70% ethanol decreases the infectivity of enveloped coronaviruses such as the canine coronavirus and the mouse hepatitis virus by 3–4 log_10_ within 10‐min exposure (Saknimit, Inatsuki, Sugiyama, & Yagami, 1988). This is of immense current significance considering the role of hand hygiene in preventing the transmission of the coronavirus disease COVID‐19 (World Health Organization, 2020). Ethanol at 40%–50% inactivates most, but not all, significant enveloped viruses within 5‐min exposure (Ciesek et al., 2010).

The available studies on the bactericidal and virucidal activity of PVP‐I demonstrate that the most favorable effect occurs at concentrations of approx. 0.08%–0.9%, with a maximum exposure of 5 min for bacteria and 60 min for the most stable viruses. The efficacy against viruses corresponds to the claim “virucidal activity/high‐level.” For PVP‐I, the carrier polyvinylpyrrolidone increases the solubility and provides a reservoir of active iodine in the aqueous medium. A chemical equilibrium develops with only about one‐thousandth part of the iodine being released and available as free molecular iodine, which is responsible for the germicidal activity (Sauerbrei & Wutzler, 2010). The most active PVP‐I concentrations with available iodine are equivalent to the free iodine concentrations in aqueous solution (Musumeki, Bandello, Martinelli, Calaresu, & Cocuzza, 2018). Lower concentrations of 0.009%–0.05% PVP‐I exert moderate bactericidal, but no virucidal/high‐level, activity. Following the decreasing free iodine concentration, the germicidal activity of PVP‐I decreases slightly, but continuously, with increasing PVP‐I concentration from 1% to 10%, resulting primarily in longer exposure times, and, especially at concentrations of 9%–10%, in partial inactivity against very stable gram‐positive cocci and poliovirus type 1 (Nishioka, Nagahama, Inoue, & Hagi, 2018; Wada et al., 2016). It is of current importance to mention that the Middle East respiratory syndrome (MERS) and the severe acute respiratory syndrome (SARS) coronaviruses are significantly inactivated by 0.23% PVP‐I within 15 s (Eggers et al., 2018), and different PVP‐I antiseptic products such as 4% PVP‐I skin cleanser, 7.5% PVP‐I surgical scrub, and 1% PVP‐I gargle/mouthwash are highly effective (Eggers, Eickmann, & Zorn, 2015).

In conclusion, the available literature data provide an overview of the bactericidal and virucidal activity of ethanol and PVP‐I in vitro determined mainly using suspension tests, and partly employing carrier tests. They can help optimize the significant inactivation of bacteria and viruses in various disciplines of medicine. However, it is a limitation of this overview that only results of in vitro tests, mainly without organic load, were included. As the conditions in application practice may differ, concrete recommendations for use can only be derived to a limited extent.

## CONFLICT OF INTEREST

None declared.

## AUTHOR CONTRIBUTIONS

**Andreas Sauerbrei:** Conceptualization (lead); data curation (lead); formal analysis (lead); validation (lead); writing – original draft (lead); writing – review & editing (lead).

## ETHICS STATEMENT

None required.

## Data Availability

All literature data associated with this article are provided in full in this paper.

## Appendix A

**TABLE A1:** Bactericidal efficacy of ethanol at concentrations of 30%–99.5%

Conc. (%)	Bacterium	Minimum time (min) for inactivation by (%)						Reference (PubMed ID)
		90	99	99.9	99.99	99.999	100	
30	*Ps. aeruginosa*	‐	‐	‐	‐	‐	30	6727697
	*Ps. cepacia*	‐	‐	‐	‐	‐	30	
	*Ps. fluorescens*	‐	‐	‐	‐	‐	1	
	*Ps. maltophilia*	‐	‐	‐	‐	‐	5	
	*Ps. putida*	‐	‐	‐	‐	‐	40 s	
	*Ps. stutzeri*	‐	‐	‐	‐	‐	20 s	
	*Fl. lutesiens*						1	
	*Fl. meningosepticum*	‐	‐	‐	‐	‐	n.e. (30 min)	
	*Acr. parvulus*	‐	‐	‐	‐	‐	2	
	*Acr. xerosis*	‐	‐	‐	‐	‐	2	
	*Acr. xylosoxidans*	‐	‐	‐	‐	‐	5	
	*Ac. calcoaceticus*	‐	‐	‐	‐	‐	30	
	*A. faecalis*	‐	‐	‐	‐	‐	2	
	*St. aureus*	‐	‐	‐	‐	‐	5	
	*St. epidermidis*	‐	‐	‐	‐	‐	30	
	*E. coli*	‐	‐	‐	‐	‐	30	
	*K. pneumoniae*	‐	‐	‐	‐	‐	5	
	*Prot. mirabilis*	‐	‐	‐	‐	‐	5	
	*Prot. morganii*	‐	‐	‐	‐	‐	5	
	*Prot. vulgaris*	‐	‐	‐	‐	‐	5	
	*En. aerogenes*	‐	‐	‐	‐	‐	5	
	*En. cloacae*	‐	‐	‐	‐	‐	5	
	*C. freundii*	‐	‐	‐	‐	‐	5	
	*S. marcescens*	‐	‐	‐	‐	‐	5	
40^1^	*Sal. Senftenberg*	n.e. (5 min)	‐	‐	‐	‐	‐	19191969
40	*Ps. aeruginosa*	‐	‐	‐	‐	‐	20 s	6727697
	*Ps. cepacia*	‐	‐	‐	‐	‐	20 s	
	*Ps. fluorescens*	‐	‐	‐	‐	‐	20 s	
	*Ps. maltophilia*	‐	‐	‐	‐	‐	20 s	
	*Ps. putida*	‐	‐	‐	‐	‐	20 s	
	*Ps. stutzeri*	‐	‐	‐	‐	‐	20 s	
	*Fl. lutesiens*						20 s	
	*Fl. meningosepticum*	‐	‐	‐	‐	‐	1	
	*Acr. parvulus*	‐	‐	‐	‐	‐	20 s	
	*Acr. xerosis*	‐	‐	‐	‐	‐	20 s	
	*Acr. xylosoxidans*	‐	‐	‐	‐	‐	20 s	
	*Ac. calcoaceticus*	‐	‐	‐	‐	‐	20 s	
	*A. faecalis*	‐	‐	‐	‐	‐	20 s	
	*St. aureus*	‐	‐	‐	‐	‐	20 s	
	*St. epidermidis*	‐	‐	‐	‐	‐	1	
	*E. coli*	‐	‐	‐	‐	‐	20 s	
	*K. pneumoniae*	‐	‐	‐	‐	‐	20 s	
	*Prot. mirabilis*	‐	‐	‐	‐	‐	20 s	
	*Prot. morganii*	‐	‐	‐	‐	‐	20 s	
	*Prot. vulgaris*	‐	‐	‐	‐	‐	20 s	
	*En. aerogenes*	‐	‐	‐	‐	‐	20 s	
	*En. cloacae*	‐	‐	‐	‐	‐	20 s	
	*C. freundii*	‐	‐	‐	‐	‐	20 s	
	*S. marcescens*	‐	‐	‐	‐	‐	20 s	
50^1^	*Sal. Senftenberg*	‐	5	‐	‐	‐	‐	19191969
56	*St. epidermidis*	‐	‐	‐	‐	‐	5	24851564
	*St. aureus*	‐	‐	‐	‐	‐	5	
	*E. coli*	‐	‐	‐	‐	‐	5	
	*Ps. aeruginosa*	‐	‐	‐	‐	‐	5	
	*K. pneumoniae*	‐	‐	‐	‐	‐	5	
60^1^	*Sal. Senftenberg*	‐	‐	‐	5	‐	‐	19191969
60	*Ps. aeruginosa*	‐	‐	‐	‐	‐	20 s	6727697
	*Ps. cepacia*	‐	‐	‐	‐	‐	20 s	
	*Ps. fluorescens*	‐	‐	‐	‐	‐	20 s	
	*Ps. maltophilia*	‐	‐	‐	‐	‐	20 s	
	*Ps. putida*	‐	‐	‐	‐	‐	20 s	
	*Ps. stutzeri*	‐	‐	‐	‐	‐	20 s	
	*Fl. lutesiens*						20 s	
	*Fl. meningosepticum*	‐	‐	‐	‐	‐	20 s	
	*Acr. parvulus*	‐	‐	‐	‐	‐	20 s	
	*Acr. xerosis*	‐	‐	‐	‐	‐	20 s	
	*Acr. xylosoxidans*	‐	‐	‐	‐	‐	20 s	
	*Ac. calcoaceticus*	‐	‐	‐	‐	‐	20 s	
	*A. faecalis*	‐	‐	‐	‐	‐	20 s	
	*St. aureus*	‐	‐	‐	‐	‐	20 s	
	*St. epidermidis*	‐	‐	‐	‐	‐	20 s	
	*E. coli*	‐	‐	‐	‐	‐	20 s	
	*K. pneumoniae*	‐	‐	‐	‐	‐	20 s	
	*Prot. mirabilis*	‐	‐	‐	‐	‐	20 s	
	*Prot. morganii*	‐	‐	‐	‐	‐	20 s	
	*Prot. vulgaris*	‐	‐	‐	‐	‐	20 s	
	*En. aerogenes*	‐	‐	‐	‐	‐	20 s	
	*En. cloacae*	‐	‐	‐	‐	‐	20 s	
	*C. freundii*	‐	‐	‐	‐	‐	20 s	
	*S. marcescens*	‐	‐	‐	‐	‐	20 s	
70^1^	*Sal. Senftenberg*	‐	‐	‐	5	‐	‐	19191969
70^2^	*Sal. spp.*	‐	‐	‐	‐	5	‐	
76.9–81.4	*Sal. spp.*	‐	‐	‐	‐	0.5		19785284
	*Sh. sonnei*	‐	‐	‐	‐	0.5		
	*Ps. aeruginosa*	‐	‐	‐	‐	0.5		
	*Pl. shigelloides*	‐	‐	‐	‐	0.5		
	*V. cholerae*	‐	‐	‐	‐	0.5		
	*Bac. subtilis*	‐	‐	‐	‐	0.5		
80	*Ps. aeruginosa*	‐	‐	‐	‐	‐	20 s	6727697
	*Ps. cepacia*	‐	‐	‐	‐	‐	20 s	
	*Ps. fluorescens*	‐	‐	‐	‐	‐	20 s	
	*Ps. maltophilia*	‐	‐	‐	‐	‐	20 s	
	*Ps. putida*	‐	‐	‐	‐	‐	20 s	
	*Ps. stutzeri*	‐	‐	‐	‐	‐	20 s	
	*Fl. lutesiens*						20 s	
	*Fl. meningosepticum*	‐	‐	‐	‐	‐	20 s	
	*Acr. parvulus*	‐	‐	‐	‐	‐	20 s	
	*Acr. xerosis*	‐	‐	‐	‐	‐	20 s	
	*Acr. xylosoxidans*	‐	‐	‐	‐	‐	20 s	
	*Ac. calcoaceticus*	‐	‐	‐	‐	‐	20 s	
	*A. faecalis*	‐	‐	‐	‐	‐	20 s	
	*St. aureus*	‐	‐	‐	‐	‐	20 s	
	*St. epidermidis*	‐	‐	‐	‐	‐	20 s	
	*E. coli*	‐	‐	‐	‐	‐	20 s	
	*K. pneumoniae*	‐	‐	‐	‐	‐	20 s	
	*Prot. mirabilis*	‐	‐	‐	‐	‐	20 s	
	*Prot. morganii*	‐	‐	‐	‐	‐	20 s	
	*Prot. vulgaris*	‐	‐	‐	‐	‐	20 s	
	*En. aerogenes*	‐	‐	‐	‐	‐	20 s	
	*En. cloacae*	‐	‐	‐	‐	‐	20 s	
	*C. freundii*	‐	‐	‐	‐	‐	20 s	
	*S. marcescens*	‐	‐	‐	‐	‐	20 s	
85^3^	*St. aureus*	‐	‐	‐	‐	0.5	‐	12392906
	*En. hirae*	‐	‐	‐	‐	0.5	‐	
	*Ps. aeruginosa*	‐	‐	‐	‐	0.5	‐	
	*E. coli*	‐	‐	‐	‐	0.5	‐	
85^3^	*Ent. faecalis*	‐	‐	‐	‐	‐	0.25	18211682
	*Ent. faecium*	‐	‐	‐	‐	‐	0.25	
	*L. monocytogenes*	‐	‐	‐	‐	‐	0.25	
	*M. luteus*	‐	‐	‐	‐	‐	0.25	
	*St. aureus*	‐	‐	‐	‐	‐	0.25	
	*St. epidermidis*	‐	‐	‐	‐	‐	0.25	
	*St haemolyticus*	‐	‐	‐	‐	‐	0.25	
	*St. hominis*	‐	‐	‐	‐	‐	0.25	
	*St. saprophyticus*	‐	‐	‐	‐	‐	0.25	
	*Str. pneumoniae*	‐	‐	‐	‐	‐	0.25	
	*Str. pyogenes*	‐	‐	‐	‐	‐	0.25	
	*Ac. baumannii*	‐	‐	‐	‐	‐	0.25	
	*Ac. lwoffi*	‐	‐	‐	‐	‐	0.25	
	*B. fragilis*	‐	‐	‐	‐	‐	0.25	
	*Bur. cepacia*	‐	‐	‐	‐	‐	0.25	
	*En. aerogenes*	‐	‐	‐	‐	‐	0.25	
	*En. cloacae*	‐	‐	‐	‐	‐	0.25	
	*E. coli*	‐	‐	‐	‐	‐	0.25	
	*H. influenzae*	‐	‐	‐	‐	‐	0.25	
	*K. pneumoniae*	‐	‐	‐	‐	‐	0.25	
	*K. oxytoca*	‐	‐	‐	‐	‐	0.25	
	*Prot. mirabilis*	‐	‐	‐	‐	‐	0.25	
	*Ps. aeruginosa*	‐	‐	‐	‐	‐	0.25	
	*Sal. enteritidis*	‐	‐	‐	‐	‐	0.25	
	*Sal. typhimurium*	‐	‐	‐	‐	‐	0.25	
	*S. marcescens*	‐	‐	‐	‐	‐	0.25	
	*Sh. sonnei*	‐	‐	‐	‐	‐	0.25	
	*Clost. difficile*	‐	‐	‐	‐	‐	0.25	
99.5	*Ps. aeruginosa*	‐	‐	‐	‐	‐	20 s	6727697
	*Ps. cepacia*	‐	‐	‐	‐	‐	20 s	
	*Ps. fluorescens*	‐	‐	‐	‐	‐	20 s	
	*Ps. maltophilia*	‐	‐	‐	‐	‐	20 s	
	*Ps. putida*	‐	‐	‐	‐	‐	20 s	
	*Ps. stutzeri*	‐	‐	‐	‐	‐	20 s	
	*Fl. lutesiens*						20 s	
	*Fl. meningosepticum*	‐	‐	‐	‐	‐	20 s	
	*Acr. parvulus*	‐	‐	‐	‐	‐	20 s	
	*Acr. xerosis*	‐	‐	‐	‐	‐	20 s	
	*Acr. xylosoxidans*	‐	‐	‐	‐	‐	20 s	
	*Ac. calcoaceticus*	‐	‐	‐	‐	‐	20 s	
	*A. faecalis*	‐	‐	‐	‐	‐	20 s	
	*St. aureus*	‐	‐	‐	‐	‐	30	
	*St. epidermidis*	‐	‐	‐	‐	‐	20 s	
	*E. coli*	‐	‐	‐	‐	‐	20 s	
	*K. pneumoniae*	‐	‐	‐	‐	‐	20 s	
	*Prot. mirabilis*	‐	‐	‐	‐	‐	20 s	
	*Prot. morganii*	‐	‐	‐	‐	‐	20 s	
	*Prot. vulgaris*	‐	‐	‐	‐	‐	20 s	
	*En. aerogenes*	‐	‐	‐	‐	‐	20 s	
	*En. cloacae*	‐	‐	‐	‐	‐	20 s	
	*C. freundii*	‐	‐	‐	‐	‐	20 s	
	*S. marcescens*	‐	‐	‐	‐	‐	20 s	

Note

No data (‐).

Abbreviations: *A*.,* Alcaligenes*;* Ac*.,* Acinetobacter*;* Acr*.,* Achromobacter*;* B*.,* Bacteroides*;* Bac*.,* Bacillus*;* Bur*.,* Burkholderia*;* C*.,* Citrobacter*;* E*.,* Escherichia*;* En*.,* Enterobacter*;* Fl*.,* Flavobacterium*;* H*.,* Haemophilus*;* K*.,* Klebsiella*;* L*.,* Listeria*;* M*.,* Micrococcus*; n.e., not effective; *Pl*.,* Plesiomonas*;* Prot*.,* Proteus*;* Ps*.,* Pseudomonas*; s, seconds; *S*.,* Serratia*;* Sal*.,* Salmonella*;* Sh*.,* Shigella*; spp., species; *St*.,* Staphylococcus*;* Str*.,* Streptococcus*;* V*.,* Vibrio*.

^1^ European surface test with bovine serum albumin.

^2^ Quantitative suspension test with bovine serum albumin.

^3^ Basic product: Sterillium Comfort Gel (85% ethanol, Bode Chemie GmbH & Co. KG, Hamburg, Germany).

**TABLE A2:** Virucidal efficacy of ethanol at concentrations of 30%–100%

Conc. (%)	Virus	Minimum time (min) for inactivation by (%)					Reference (PubMed ID)
		90	99	99.90	99.99	100	
30	BRV	‐	1	‐	‐	‐	6182233
	FCV	n.e. (10 min)	‐	‐	‐	‐	16443090
	MNV	n.e. (3 min)	‐	‐	‐	‐	18378650
	BVDV	1	‐	5	‐	‐	20441517
	HCV	5	‐	‐	‐	‐	
	VV	n.e. (1 min)	‐	‐	‐	‐	20573218
	MVA	n.e. (1 min)	‐	‐	‐	‐	
	HCV	n.e. (5 min)	‐	‐	‐	‐	22013220
	DHBV	‐	‐	2	‐	‐	23110658
	VV	n.e. (2 min)	‐	‐	‐	‐	
40	BRV	‐	‐	‐	1	‐	6182233
	CV‐A16	‐	‐	‐	‐^a^	‐	6274971
	EV‐71	‐	‐	‐	‐^a^	‐	
	ECHO‐7	‐	‐	‐	‐^a^	‐	
	PV‐1	‐	‐	‐	‐^a^	‐	
	CV‐B5	‐	‐	‐	‐^a^	‐	
	EV‐70	‐	‐	‐	‐^a^	‐	
	AV‐3	‐	‐	‐	‐^a^	‐	
	VV	‐	‐	‐	0.5^b^	‐	
	IVA	‐	‐	‐	1^b^	‐	
	NDV	‐	‐	‐	10 s^b^	‐	
	HSV	‐	‐	‐	10 s^b^	‐	
	FCV	1	3	‐	‐	‐	16443090
	MNV^c^	n.e. (5 min)	‐	‐	‐	‐	19583832
	BVDV	‐	‐	‐	1	‐	20441517
	HCV	1	5	‐	‐	‐	
	VV	‐	1	‐	‐	‐	20573218
	MVA	‐	1	‐	‐	‐	
	HCV	1	‐	‐	‐	‐	22013220
40	DHBV	‐	‐	‐	1	‐	23110658
	VV	‐	‐	‐	1	‐	
50	CV‐A16	‐	‐	‐	‐^a^	‐	6274971
	EV‐71	‐	‐	‐	‐^a^	‐	
	ECHO‐7	‐	‐	‐	‐^a^	‐	
	PV‐1	‐	‐	‐	‐^a^	‐	
	CV‐B5	‐	‐	‐	‐^a^	‐	
	EV‐70	‐	‐	‐	0.5^b^	‐	
	AV‐3	‐	‐	‐	‐^a^	‐	
	VV	‐	‐	‐	10 s^b^	‐	
	IVA	‐	‐	‐	10 s^b^	‐	
	NDV	‐	‐	‐	10 s^b^	‐	
	HSV	‐	‐	‐	10 s^b^	‐	
	FCV	‐	0.5	1	3	‐	14706271
	FCV	10	‐	‐	‐	‐	16443090
	MNV	‐	‐	‐	5	‐	19583832
	VV	‐	‐	‐	1	‐	20573218
	MVA	‐	‐	‐	1	‐	
	HCV	‐	‐	‐	5	‐	22013220
	MNV	‐	‐	0.5	‐	‐	21862176
	DHBV	‐	‐	‐	1	‐	23110658
	VV	‐	‐	‐	1	‐	
	NoV‐VLP	‐	‐	‐	‐	1	27554301
60	ECHO‐11	n.e. (1 min)	‐	‐	‐	‐	6182233
	CV‐A16	‐	‐	‐	‐^a^	‐	6274971
	EV‐71	‐	‐	‐	‐^a^	‐	
	ECHO‐7	‐	‐	‐	‐^a^	‐	
	PV‐1	‐	‐	‐	2^b^	‐	
	CV‐B5	‐	‐	‐	2^b^	‐	
	EV‐70	‐	‐	‐	0.5^b^	‐	
	AV‐3	‐	‐	‐	‐^a^	‐	
	VV	‐	‐	‐	10 s^b^	‐	
	IVA	‐	‐	‐	10 s^b^	‐	
	NDV	‐	‐	‐	10 s^b^	‐	
	HSV	‐	‐	‐	10 s^b^	‐	
	FCV	10	‐	‐	‐	‐	16443090
60	MNV	‐	‐	‐	0.5	‐	18378650
	MNV	‐	‐	‐	5	‐	19583832
	VV	‐	‐	‐	1	‐	20573218
	MVA	‐	‐	‐	1	‐	
	FCV	1	‐	‐	‐	‐	19616346
	HCV	‐	‐	‐	1	‐	22013220
	MNV	‐	‐	‐	0.5	‐	21862176
	DHBV	‐	‐	‐	1	‐	23110658
	VV	‐	‐	‐	1	‐	
	NoV‐VLP	‐	‐	‐	‐	0.5	27554301
68^1^	OPV	‐	‐	‐	0.25	‐	12392906
	HSV‐1/2	‐	‐	‐	0.25	‐	
	AV‐2	‐	‐	‐	2	‐	
	PV‐1	‐	‐	‐	3	‐	
	PolyV SV‐40	‐	‐	‐	15	‐	
	ROV	‐	‐	‐	0.5	‐	
	HIV	‐	‐	‐	0.5	‐	
70	ASV	‐	‐	‐	1	‐	6182233
	CV‐A16	‐	‐	‐	‐^a^	‐	6274971
	EV‐71	‐	‐	‐	‐^a^	‐	
	ECHO‐7	‐	‐	‐	‐^a^	‐	
	PV‐1	‐	‐	‐	1^b^	‐	
	CV‐B5	‐	‐	‐	1^b^	‐	
	EV‐70	‐	‐	‐	0.5^b^	‐	
	AV‐3	‐	‐	‐	‐^a^	‐	
	VV	‐	‐	‐	10 s^b^	‐	
	IVA	‐	‐	‐	10 s^b^	‐	
	NDV	‐	‐	‐	10 s^b^	‐	
	HSV	‐	‐	‐	10 s^b^	‐	
	HSV‐1	‐	‐	‐	1	‐	2880894
	CPV	10	‐	‐	‐	‐	3416941
	KRV	10	‐	‐	‐	‐	
	MHV	‐	‐	‐	10	‐	
	CCoV	‐	‐	10	‐	‐	
	PV‐1	‐	1	‐	10	‐	1972949^d^
	FCV	1	4	30	60	‐	15294783
	CCV	1	16	‐	60	‐	
	FCV	‐	‐	0.5	3	‐	14706271
	FCV	‐	1	‐	‐	‐	16443090
	PPV	n.e. (10 min)	‐	‐	‐	‐	19646784
	MVM	n.e. (10 min)	‐	‐	‐	‐	
70	PV‐1	10	1	‐	‐	‐	19646784
	AV‐5	‐	1	‐	10	‐	
	VV	‐	‐	‐	1	‐	
	PV‐1	n.e. (30 min)	‐	‐	‐	‐	19482374
	ECHO‐1	5	‐	‐	10	‐	
	MNV	‐	‐	‐	0.5	‐	21862176
	NoV‐VLP	‐	‐	‐	‐	0.5	27554301
>72	FCV	n.e. (0.5 min)	‐	‐	‐	‐	23009803
	MNV	‐	‐	‐	0.5	‐	
	AV‐5	‐	‐	‐	0.5	‐	
	PV‐1	‐	‐	‐	0.5	‐	
	VV	‐	‐	‐	0.5	‐	
75	FCV	1	‐	‐	‐	‐	9949965
76	ECHO‐11	‐	‐	1	‐	‐	6182233
80	CV‐A16	‐	‐	‐	‐^a^	‐	6274971
	EV‐71	‐	‐	‐	1^b^	‐	
	ECHO‐7	‐	‐	‐	1^b^	‐	
	PV‐1	‐	‐	‐	0.5^b^	‐	
	CV‐B5	‐	‐	‐	0.5^b^	‐	
	EV‐70	‐	‐	‐	10 s^b^	‐	
	AV‐3	‐	‐	‐	2^b^	‐	
	VV	‐	‐	‐	10 s^b^	‐	
	IVA	‐	‐	‐	10 s^b^	‐	
	NDV	‐	‐	‐	10 s^b^	‐	
	HSV	‐	‐	‐	10 s^b^	‐	
	PV‐1	‐	1	‐	10	‐	1972949^d^
	FCV	‐	0.5	3	5	‐	14706271
	FCV	1	‐	‐	‐	‐	16443090
	PV‐1	5	10	‐	‐	‐	19482374
	ECHO‐1	‐	2	5	10	‐	
	MNV	‐	‐	‐	0.5	‐	21862176
90	ASV	‐	‐	‐	1	‐	6182233
	CV‐A16	‐	‐	‐	5^b^	‐	6274971
	EV‐71	‐	‐	‐	0.5^b^	‐	
	ECHO‐7	‐	‐	‐	0.5^b^	‐	
	PV‐1	‐	‐	‐	10 s^b^	‐	
	CV‐B5	‐	‐	‐	10 s^b^	‐	
	EV‐70	‐	‐	‐	10 s^b^	‐	
	AV‐3	‐	‐	‐	0.5^b^	‐	
	VV	‐	‐	‐	10 s^b^	‐	
	IVA	‐	‐	‐	10 s^b^	‐	
	NDV	‐	‐	‐	10 s^b^	‐	
	HSV	‐	‐	‐	10 s^b^	‐	
	HSV‐1	‐	‐	‐	5	‐	2880894
	PV‐1	1	‐	‐	‐	‐	1972949^d^
	FCV	‐	1	‐	‐	‐	16443090
	MNV	‐	‐	‐	0.5	‐	21862176
95	HSV‐1	5	10	‐	‐	‐	2880894
	HAV	‐	‐	2	‐	‐	11759019
100	HSV‐1	10	‐	‐	‐	‐	2880894
	PV‐1	1	5	‐	‐	‐	1972949^d^
	FCV	1	‐	‐	‐		16443090

Note

No data (‐).

Abbreviations: ASV, astrovirus; AV‐3, adenovirus type 3; AV‐5, adenovirus type 5; BVDV, bovine viral diarrhea virus; BRV, bovine rotavirus; CCV, canine calicivirus; CCoV, canine coronavirus; CPV, canine parvovirus; CV‐A16, coxsackievirus A16; CV‐B5, coxsackievirus B5; DHBV, duck hepatitis B virus; ECHO‐1, ECHO virus type 1; ECHO‐7, ECHO virus type 7; ECHO‐11, ECHO virus type 11; EV‐70, enterovirus 70; EV‐71, enterovirus 71; FCV, feline calicivirus; HAV, hepatitis A virus; HCV, hepatitis C virus; HIV, human immunodeficiency virus; HSV‐1/2, herpes simplex virus type 1/2; IAV, influenza A virus; KRV, Kilham rat virus; MHV, mouse hepatitis virus; MNV, murine norovirus; MVA, modified vaccinia virus Ankara; MVM, minute virus of mice; NDV, Newcastle disease virus; n.e., not effective; NoV‐VLP, norovirus‐like particles; OPV, orthopoxvirus; PAPV, papovavirus; PolyV, polyomavirus; PPV, porcine parvovirus; PV‐1, poliovirus type 1; ROV, rotavirus; s, seconds; VV, vaccinia virus.

^a^ No perfect inactivation.

^b^ Perfect inactivation.

^c^ Exposure time 5 min.

^d^ Carrier test.

^1^ Basic product: Sterillium Gel (Bode Chemie GmbH & Co. KG, Hamburg, Germany).

**TABLE A3:** Bactericidal efficacy of PVP‐I at concentrations of 0.009%–10%

Conc. (%)	Bacterium	Minimum time (min) for inactivation by (%)						Reference (PubMed ID)
		90	99	99.9	99.99	99.999	100	
0.009^1^	MRSA	‐	‐	0.25	‐	‐	0.5	4008627
	MSSA	‐	‐	0.25	‐	‐	0.5	
	*E. coli*	‐	‐	5	‐	‐	15	21168786
0.01^2^	MRSA spp.	‐	0.5	5	‐	‐	‐	9531717
	*Ent. faecium*	0.5	1	‐	‐	‐	‐	
	MRSA	‐	0.5	5	‐	‐	‐	12011534
	*Ent. faecium*	0.5	1	‐	‐	‐	‐	
0.011^2^	*Cl. trachomatis*	5	‐	‐	‐	‐	‐	10754445
0.02	*Myc. avium*	‐	‐	‐	‐	‐	0.5	10864189
	*Myc. kansasii*	‐	‐	‐	‐	‐	1	
	*Myc. tuberculosis*	‐	‐	‐	‐	‐	0.5	
0.023^2^	*Cl. trachomatis*	‐	‐	‐	‐	0.5	‐	10754445
0.036^3^	*Str. mutans*	‐	‐	0.5	‐	‐	‐	9566143
	*Por. gingivalis*	‐	‐	‐	‐	‐	10 s	
	*Prev. intermedia*	‐	‐	‐	‐	‐	10 s	
	MRSA	‐	‐	‐	‐	‐	0.5	
	*Str. pyogenes*	‐	‐	‐	‐	‐	10 s	
	*Hel. pylori*	n.e. (0.5 min)	‐	‐	‐	‐	‐	
0.045^2^	*Cl. trachomatis*	‐	‐	‐	‐	0.5	‐	10754445
0.05^4^	MRSA spp.	‐	‐	‐	‐	‐	1	10896798
	MSSA spp.	‐	‐	‐	‐	‐	1	
0.05^5^	MRSA spp.	‐	‐	‐	‐	0.5	‐	1355784
0.05^6^, ^7^	*Bord. pertussis*	‐	‐	‐	‐	0.25	‐	21968967
0.07^8^	*K. pneumoniae*	‐	‐	0.5	‐	‐	‐	29633177
	*Str. pneumoniae*	‐	‐	‐	0.25	‐	‐	
0.07^2^	*Sal. spp.*	‐	‐	‐	‐	0.5	‐	19785284
	*Sh. sonnei*	‐	‐	‐	‐	0.5	‐	
	*Ps. aeruginosa*	‐	‐	‐	‐	0.5	‐	
	*Pl. shigelloides*	‐	‐	‐	‐	0.5	‐	
	*V. cholerae*	‐	‐	‐	‐	0.5	‐	
	*Bac. subtilis*	‐	‐	‐	0.5	‐	‐	
0.09^2^	*Cl. trachomatis*	‐	‐	‐	‐	0.5	‐	10754445
0.09^10^	*E. coli*	‐	‐	‐	‐	5	5	21168786
0.09^1^	MRSA spp.	‐	‐	‐	‐	‐	0.25	4008627
	MSSA spp.	‐	‐	‐	‐	‐	0.25	
0.09^10^	*St. aureus*	‐	‐	‐	‐	‐	0.25	7040461
	*Myc. chelonei*	‐	‐	‐	‐	‐	0.5	
	*K. pneumoniae*	‐	‐	‐	‐	‐	0.25	
	*Ps. cepacia*	‐	‐	‐	‐	‐	0.25	
	*Str. mitis*	‐	‐	‐	‐	‐	0.25	
0.1^11^	*Ps. aeruginosa*	‐	1	‐	‐	‐	‐	25779009
	*E. coli*	‐	‐	‐	‐	1	‐	
	*St. aureus*	‐	n.e. (1 min)	‐	‐	‐	‐	
	*Ent. hirae*	n.e. (1 min)	‐	‐	‐	‐	‐	
0.1^5^	MRSA spp.	‐	‐	‐	‐	0.5	‐	1355784
0.1	*Myc. avium*	‐	‐	‐	‐	‐	0.5	10864189
	*Myc. kansasii*	‐	‐	‐	‐	‐	0.5	
	*Myc. tuberculosis*	‐	‐	‐	‐	‐	0.5	
	*Myc*.* tuberculosis spp*.	‐	‐	1	‐	‐	‐	12234131
0.1^2^	MRSA spp.	‐	‐	‐	‐	0.5	‐	9531717
	*Ent. faecium*	‐	‐	‐	‐	0.5	‐	
	MRSA	‐	‐	‐	‐	0.5	‐	12011534
	*Ent. faecium*	‐	‐	‐	‐	0.5	‐	
0.1^6^	*St. aureus*	‐	‐	‐	‐	0.5	‐	12011519
	MSSA	‐	‐	‐	‐	0.5	‐	
	MRSA	‐	‐	‐	‐	0.5	‐	
	*Ps. aeruginosa spp.*	‐	‐	‐	‐	0.5	‐	
	*K. pneumoniae spp.*	‐	‐	‐	‐	0.5	‐	
0.18^2^	*Cl. trachomatis*	‐	‐	‐	‐	0.5	‐	10754445
0.18^10^	*St. aureus*	‐	‐	‐	‐	‐	0.25	7040461
	*Myc. chelonei*	‐	0.5	‐	‐	‐	1	
	*K. pneumoniae*	‐	‐	‐	‐	‐	0.25	
	*Ps. cepacia*	‐	‐	‐	‐	‐	0.25	
	*Str. mitis*	‐	‐	‐	‐	‐	0.25	
0.2^5^	MRSA spp.	‐	‐	‐	‐	0.5	‐	1355784
0.2^9^	*S. marcescens*	‐	‐	‐	‐	‐	0.5	12011516
	*Ps. aeruginosa*	‐	‐	‐	‐	‐	0.5	
	*K. pneumonia*	‐	‐	‐	‐	‐	0.5	
	*A. faecalis*	‐	‐	‐	‐	‐	0.5	
	*A. xylosoxydans*	‐	‐	‐	‐	‐	0.5	
0.2	*Myc. tuberculosis spp.*	‐	‐	‐	‐	‐	2	12234131
0.2^6^, ^7^	*Bord. pertussis*	‐	‐	‐	‐	0.25	‐	21968967
0.21^6^	*St. aureus*	‐	‐	‐	‐	0.5	‐	12011519
	MSSA	‐	‐	‐	‐	0.5	‐	
	MRSA	‐	‐	‐	‐	0.5	‐	
	*Ps. aeruginosa spp.*	‐	‐	‐	‐	0.5	‐	
	*K. pneumoniae spp.*	‐	‐	‐	‐	0.5	‐	
0.23^8^	*K. pneumoniae*	‐	‐	‐	‐	0.25	‐	29633177
	*Str. pneumoniae*	‐	‐	‐	‐	0.25	‐	
0.23^6^	*Por. gingivalis spp.*	‐	‐	‐	‐	‐	0.25	16490986
	*Act. actinomycetem‐comitans spp.*	‐	‐	‐	‐	‐	0.25	
	*F. nucleatum*	‐	‐	‐	‐	‐	0.25	
	*T. forsythensis*	‐	‐	‐	‐	‐	0.25	
	*Prev. intermedia*	‐	‐	‐	‐	‐	0.25	
	*Str. anginosus*	‐	‐	‐	‐	‐	0.25	
0.4^5^	MRSA	‐	‐	‐	‐	0.5	‐	1355784
0.42^6^	*St. aureus*	‐	‐	‐	‐	0.5	‐	12011519
	MSSA	‐	‐	‐	‐	0.5	‐	
	MRSA	‐	‐	‐	‐	0.5	‐	
	*Ps. aeruginosa spp.*	‐	‐	‐	‐	0.5	‐	
	*K. pneumoniae spp.*	‐	‐	‐	‐	0.5	‐	
0.47^6^	*Por. gingivalis spp.*	‐	‐	‐	‐	‐	0.25	16490986
	*Act. actinomycetem‐comitans spp.*	‐	‐	‐	‐	‐	0.25	
	*F. nucleatum*	‐	‐	‐	‐	‐	0.25	
	*T. forsythensis*	‐	‐	‐	‐	‐	0.25	
	*Prev. intermedia*	‐	‐	‐	‐	‐	0.25	
	*Str. anginosus*	‐	‐	‐	‐	‐	0.25	
0.5^4^	MRSA spp.	‐	‐	‐	‐	‐	1	10896798
	MSSA spp.	‐	‐	‐	‐	‐	1	
0.5^6^, ^7^	*Bord. pertussis*	‐	‐	‐	‐	0.25	‐	21968967
0,57^12^	MSSA	‐	‐	‐	‐	‐	1	30295039
	MRSA	‐	‐	‐	‐	‐	1	
	MSSE	‐	‐	‐	‐	‐	1	
	MRSE	‐	‐	‐	‐	‐	1	
	*Ps. aeruginosa*	‐	‐	‐	‐	‐	2	
	*E. coli*	‐	‐	‐	‐	‐	2	
0.625	MSSA	‐	‐	‐	‐	120	‐	21035920
	MRSA	‐	‐	‐	‐	120	‐	
	MRSE	‐	‐	‐	‐	120	‐	
	*Ac. baumannii*	‐	‐	‐	‐	120	‐	
	*Ps. aeruginosa*	‐	‐	‐	‐	120	‐	
	*E. coli*	‐	‐	‐	‐	120	‐	
0.7^8^	*K. pneumoniae*	‐	‐	‐	‐	0.25	‐	29633177
	*Str. pneumoniae*	‐	‐	‐	‐	0.25	‐	
0.7^9^	*Sal. spp.*	‐	‐	‐	‐	0.5	‐	19785284
	*Sh. sonnei*	‐	‐	‐	‐	0.5	‐	
	*Ps. aeruginosa*	‐	‐	‐	‐	0.5	‐	
	*Pl. shigelloides*	‐	‐	‐	‐	0.5	‐	
	*V. cholerae*	‐	‐	‐	‐	0.5	‐	
	*Bac. subtilis*	‐	0.5	‐	‐	‐	‐	
0.9	*St. aureus spp.*	‐	‐	‐	‐	‐	2	2368748
0.9^1^	MRSA spp.	‐	0.25	‐	‐	‐	0.5	4008627
	MSSA spp.	‐	‐	‐	‐	‐	0.25	
0,9^10^	*St. aureus*	‐	‐	‐	‐	‐	0.25	7040461
	*Myc. chelonei*	1	‐	‐	‐	‐	2	
	*K. pneumoniae*	‐	‐	‐	‐	‐	0.25	
	*Ps. cepacia*	‐	‐	‐	‐	‐	0.25	
	*Str. mitis*	‐	‐	‐	‐	‐	0.25	
1.0^13^	*St. aureus*	‐	‐	‐	‐	‐	30	3238890
1.0^11^	*Ps. aeruginosa*	‐	‐	‐	‐	1	‐	25779009
	*E. coli*	‐	‐	‐	‐	1	‐	
	*St. aureus*	‐	‐	‐	‐	1	‐	
	*Ent. hirae*	n.e. (1 min)	‐	‐	‐	‐	‐	
1.0^2^	MRSA spp.	‐	‐	‐	‐	0.5	‐	9531717
	*Ent. faecium*	‐	‐	‐	0.5	1	‐	
	MRSA	‐	‐	‐	‐	0.5	‐	12011534
	*Ent. faecium*	‐	‐	‐	0.5	1	‐	
1.8	*Ps. aeruginosa*	‐	‐	‐	1	‐	‐	11232776
	*Ent. faecium*	1	‐	‐	‐	‐	‐	
	*St. epidermidis*	‐	1	‐	‐	‐	‐	
	*St. aureus*	‐	‐	‐	1	‐	‐	
	MRSA	‐	‐	‐	1	‐	‐	
	*E. coli*	‐	‐	‐	1	‐	‐	
	*Ent. faecalis*	‐	1	‐	‐	‐	‐	
2.0^14^	*Ps. aeruginosa*	1	‐	‐	‐	‐	‐	11232776
	*Ent. faecium*	1	‐	‐	‐	‐	‐	
	*St. epidermidis*	1	‐	‐	‐	‐	‐	
	*St. aureus*	1	‐	‐	‐	‐	‐	
	MRSA	1	‐	‐	‐	‐	‐	
	*E. coli*	1	‐	‐	‐	‐	‐	
	*Ent. faecalis*	1	‐	‐	‐	‐	‐	
2.0^15^	*Ps. aeruginosa*	n.e. (1 min)	‐	‐	‐	‐	‐	11232776
	*Ent. faecium*	n.e. (1 min)	‐	‐	‐	‐	‐	
	*St. epidermidis*	n.e. (1 min)	‐	‐	‐	‐	‐	
	*St. aureus*	n.e. (1 min)	‐	‐	‐	‐	‐	
	MRSA	n.e. (1 min)	‐	‐	‐	‐	‐	
	*E. coli*	n.e. (1 min)	‐	‐	‐	‐	‐	
	*Ent. faecalis*	n.e. (1 min)	‐	‐	‐	‐	‐	
2.3^10^	*St. aureus*	‐	0.25	‐	‐	‐	0.5	7040461
	*Myc. chelonei*	‐	2	‐	‐	‐	4	
	*K. pneumoniae*	‐	‐	‐	‐	‐	0.25	
	*Ps. cepacia*	‐	‐	‐	‐	‐	0.25	
	*Str. mitis*	‐	‐	‐	‐	‐	0.25	
2.5^16^	*St. aureus*	‐	‐	1	‐	5	‐	11096195
2.5^17^	MSSA	‐	‐	1	‐	‐	‐	29985866
	MSSE	‐	‐	1	‐	‐	‐	
	MRSA	‐	‐	1	‐	‐	‐	
	MRSE	‐	‐	0.25	‐	‐	‐	
	*Cory. species*	‐	‐	0.25	‐	‐	‐	
	*Pr. acnes*	‐	‐	0.25	‐	‐	‐	
	*Ps. aeruginosa*	‐	‐	0.25	‐	‐	‐	
	*Str. pyogenes*	‐	‐	0.25	‐	‐	‐	
	*St. capitis*	‐	‐	1	‐	‐	‐	
	*St. xylosus*	‐	‐	2	‐	‐	‐	
4.6^10^	*St. aureus*	0.25	0.5	‐	‐	‐	1	7040461
	*Myc. chelonei*	‐	‐	‐	‐	‐	4	
	*K. pneumoniae*	‐	‐	‐	‐	‐	0.25	
	*Ps. cepacia*	‐	‐	‐	‐	‐	0.25	
	*Str. mitis*	‐	‐	‐	‐	‐	0.25	
5.75^18^	MSSA	‐	‐	‐	‐	‐	2	30295039
	MRSA	‐	‐	‐	‐	‐	4	
	MSSE	‐	‐	‐	‐	‐	4	
	MRSE	‐	‐	‐	‐	‐	6	
	*Ps. aeruginosa*	‐	‐	‐	‐	‐	6	
	*E. coli*	‐	‐	‐	‐	‐	4	
5.0^16^	*St. aureus*	1	‐	5	‐	15	‐	11096195
5.0^11^	*Ps. aeruginosa*	‐	‐	‐	‐	1	‐	25779009
	*E. coli*	‐	‐	‐	‐	1	‐	
	*St. aureus*	‐	‐	‐	‐	1	‐	
	*Ent. hirae*	n.e. (1 min)	‐	‐	‐	‐	‐	
5.0^13^	*St. aureus*	‐	‐	‐	‐	‐	30	3238890
6.9^19^	*St. aureus*	‐	‐	‐	‐	0.25	‐	16650702
	*Ps. aeruginosa*	‐	‐	‐	‐	0.25	‐	
7.4^16^	*St. aureus*	5	‐	15	‐	30	‐	11096195
9.0^13^	*St. aureus spp.*	‐	‐	‐	‐	‐	4	2368748
9.1^1^	MRSA spp.	0.25	0.5	1	‐	‐	2	4008627
	MSSA spp.	‐	0.25	‐	‐	‐	0.5	
9.1^10^	*St. aureus*	0.5	1	2	‐	‐	4	7040461
	*Myc. chelonei*	4	‐	‐	‐	‐	8	
	*K. pneumoniae*	‐	‐	‐	‐	‐	0.25	
	*Ps. cepacia*	‐	‐	‐	‐	‐	0.25	
	*Str. mitis*	‐	‐	‐	‐	‐	0.25	
9.7^11^	*Ps. aeruginosa*	‐	‐	‐	‐	1	‐	25779009
	*E. coli*	‐	‐	‐	‐	1	‐	
	*St. aureus*	‐	n.e. (1 min)	‐	‐	‐	‐	
	*Ent. hirae*	n.e. (1 min)	‐	‐	‐	‐	‐	
9.9^2^	MRSA spp.	‐	‐	‐	‐	0.5	‐	9531717
	*Ent. faecium*	‐	0.5	1	‐	5		
	MRSA	‐	‐	‐	‐	0.5	‐	12011534
	*Ent. faecium*	‐	0.5	1	‐	5	‐	
9.9^20^	*St. aureus*	0.25	‐	0.5	‐	1	‐	16650702
	*Ps. aeruginosa*	‐	‐	‐	‐	0.25	‐	
9.9^21^	*St. aureus*	‐	‐	‐	‐	0.5	‐	4022760
	*Ps. aeruginosa*	‐	‐	‐	‐	0.5	‐	
9.9	*St. epidermidis*	‐	‐	‐	‐	0.5		16221509
	*St. aureus*	‐	‐	‐	‐	5		29897541
	MRSA	‐	‐	‐	‐	1		
	*Str. pyogenes*	‐	‐	‐	‐	1		
	*Ent. faecalis*	‐	‐	‐	‐	5		
	*E. coli*	‐	‐	‐	‐	1		
	*Ps. aeruginosa*	‐	‐	‐	‐	1		
	*K. pneumoniae*	‐	‐	‐	‐	1		
	*Bac. cereus*	‐	‐	‐	‐	60		
	*Ac. baumannii*	‐	‐	‐	‐	1	‐	
9.9^16^	*St. aureus*	30	‐	‐	‐		‐	11096195
10.0^15^, ^22^	MRSA	0.5	3	‐	‐	‐	‐	30403371
	*St. epidermidis*	‐	0.5	3	‐	‐	‐	
	*Ent. faecalis*	n.e. (3 min)	‐	‐	‐	‐	‐	
	*Ac. baumannii*	‐	‐	0.5	‐	‐	‐	
	*Cory. minutissimum*	‐	0.5	3	‐	‐	‐	
	*Cu. acnes*	‐	‐	0.5	‐	‐	‐	
10.0	*St. aureus*	‐	‐	‐	‐	5	‐	28193164
	*Ent. faecium*	‐	‐	‐	‐	30	‐	
	*Ps. aeruginosa*	‐	‐	‐	‐	5	‐	

Note

No data (‐).

Abbreviations: *A*.,* Alcaligenes*; *Ac*.,* Acinetobacter*; *Act*.,* Actinobacillus*; *Bac*.,* Bacillus*; *Bord*.,* Bordetella*; *Cl*.,* Chlamydia*; *Cory*.,* Corynebacterium*; *Cu*.,* Cutibacterium*; *E*.,* Escherichia*; *Ent*.,* Enterococcus*; *F*.,* Fusobacterium*; *Hel*.,* Helicobacter*;* K*.,* Klebsiella*;* Myc*.,* Mycobacterium*; MRSA, Methicillin‐resistant *Staphylococcus aureus*; MRSE, Methicillin‐resistant *Staphylococcus epidermidis*; MSSA, Methicillin‐susceptible *Staphylococcus aureus*; MSSE, Methicillin‐susceptible *Staphylococcus epidermidis*; n.e., not effective; *Pl*.,* Plesiomonas*;* Por*.,* Porphyromonas*;* Pr*.,* Propionibacterium*;* Prev*.,* Prevotella*;* Ps*.,* Pseudomonas*; s, seconds; *S*.,* Serratia*;* Sal*.,* Salmonella*;* Sh*.,* Shigella*; spp., species; *St*.,* Staphylococcus*;* Str*.,* Streptococcus*;* T*.,* Tannerella*;* V*.,* Vibrio*.

^1^ Basic product: Betadine (10% PVP‐I, Purdue Frederick Co., Stamford, CT, USA).

^2^ Basic product: Betaisodona^®^ (10% PVP‐I, Mundipharma, Limburg, Germany).

^3^ Basic product: Isodine^®^ (2% PVP‐I, Meiji Seika Kaisha Ltd., Tokyo, Japan).

^4^ Basic product: Betadine Cream (5% PVP‐I, Seton Healthcare Ltd., Oldham, UK).

^5^ Basic product: Betadine Antiseptic Solution (10% PVP‐I, Napp Laboratories, Cambridge, UK).

^6^ Basic product: Isodine^®^ Gargle (7% Meiji Seika Kaisha Ltd., Tokyo, Japan).

^7^ Basic product: Isodine^®^ solution (10% PVP‐I, Meiji Seika Kaisha Ltd., Tokyo, Japan).

^8^ Basic product: Isodine^®^ (7% PVP‐I, Fukuchi Pharmaceutical Co, Ltd., Hinocho Gamou‐Gun, Japan).

^9^ Basic product: Isodine^®^ (7% PVP‐I, Meiji Seika Kaisha Ltd., Tokyo, Japan).

^10^ Basic product: Povidine (10% PVP‐I, National Pharmaceutical Manufacturing Co, Washington, DC, USA).

^11^ Basic product: Dermal Betadine^®^ (10% PVP‐I), Purdue Frederick Co., Stamford, CT, USA).

^12^ Basic product: IODIM^®^ (0.6% PVP‐I, Medivis Srl, Catania, Italy).

^13^ Basic product: Betadine^®^ (10% PVP‐I, Purdue Frederick Co., Stamford, CT, USA).

^14^ Glas carrier test.

^15^ Ex‐vivo skin test.

^16^ Basic product: PVP‐I‐Salbe (10% PVP‐I, Mundipharma, Limburg, Germany).

^17^ Basic product: Betadine (5% PVP‐I, Alcon Laboratories, Inc., Fort Worth, TX, USA).

^18^ Basic product: Oftasteril^®^ (5% PVP‐I, Alfa Intes Srl, Casoria, Italy).

^19^ Basic product: Braunol^®^ (7.5% PVP‐I, B. Braun Medical, Melsungen, Germany).

^20^ Basic product: Betadine^®^ (10% PVP‐I, Mundipharma, Basel, Switzerland).

^21^ Basic product: iso‐Betadine dermicum^®^ (10% PVP‐I, Belgana, Brussels, Belgium).

^22^ Basic product: Isodine^®^ solution 10% (10% PVP‐I, Mundipharma KK, Tokyo, Japan).

**TABLE A4:** Virucidal efficacy of PVP‐I at concentrations of 0.008%–10%

Conc. (%)	Virus	Minimum time (min) for inactivation by (%)				Reference (PubMed ID)
		90	99	99.90	99.99	
0.008	PV‐1	n.e. (5 min)	‐	‐	‐	9403252
	CV‐B3	‐	‐	3	5	
	PV‐3	‐	‐	0.5	1	
0.009^1^	IAV	‐	‐	‐	0.5	12062394
0.009	IAV	‐	‐	‐	0.25	27009506
	PV‐1	5	‐	15	30	
	AV‐3	0.25	‐	1	5	
0.023^2^	IAV	‐	‐	‐	0.25	29633177
	ROV	0.25	0.5	‐	‐	
0.025	HIV	‐	‐	0.5	‐	9403252
0.03	PV‐1	0.5	1	5	‐	
	CV‐B3	1	‐	‐	3	
	PV‐3	‐	‐	0.5	1	
0.05^1^	DHBV	n.e. (15 min)	‐	‐	‐	17011665
0.05^3^	AV‐5	0.5	60	‐	‐	15142717
	AV‐26	0.5	5	‐	60	
	AV‐44	2	‐	‐	‐	
0.05	AV‐5	0.25	‐	‐	‐	9403252
	HSV‐1	‐	‐	‐	0.25	
	RV	0.5	‐	‐	‐	
	MV	‐	‐	‐	0.5	
	IVA	n.e. (10 min)	‐	‐	‐	
	ROV	n.e. (10 min)	‐	‐	‐	
	HRV	‐	‐	0.25	‐	
	HIV	‐	‐	0.5	‐	
0.0625	PV‐1	‐	0.5	5	‐	
0.08^4^	MVA	‐	‐	‐	0.25	26381737
	EBOV	‐	‐	‐	0.25	
0.09^1^	HSV‐1	‐	‐	‐	0.5	10754445
	AV‐8	1	2	‐	5	
0.09	IAV	‐	‐	‐	0.25	27009506
	PV‐1	‐	5	‐	15	
	AV‐3	‐	0.25	‐	1	
0.1	AV‐5	‐	0.25	‐	‐	9403252
	HSV‐1	‐	‐	‐	0.25	
	MAV	‐	‐	‐	0.5	
	IVA	0.25	‐	‐	‐	
	ROV	‐	0.25	‐	‐	
	HRV	‐	0.25	1	‐	
	HIV	‐	‐	0.5	‐	
0.11^1^	HSV‐1	‐	‐	‐	0.5	12062394
0.125^1^	DHBV	15	‐	‐	‐	17011665
0.125^3^	AV‐5	0.5	15	60	‐	15142717
	AV‐26	‐	0.5	‐	2	
	AV‐44	0.5	‐	‐	60	
0.125	PV‐1	0.5	‐	5	‐	9403252
	CV‐B3	‐	‐	3	5	
	PV‐3	‐	0.5	1	3	
	IAV	‐	‐	‐	10 s	16490988
0.2^5^	MNV	‐	‐	‐	0.25	22293670
0.225^1^	HSV‐1	‐	‐	‐	0.5	10754445
	AV‐8	1	2	‐	5	
0.23^1^	AV‐8	0.5	1.5	‐	5	12062394
0.23^2^	IAV	‐	‐	‐	0.25	29633177
	SARS‐CoV	‐	‐	‐	0.25	
	MERS‐CoV	‐	‐	‐	0.25	
	ROV	‐	‐	‐	0.25	
0.25	IAV	‐	‐	‐	10 s	16490988
0.4	AV‐3	1	‐	‐	5	30589605
	AV‐4	‐	1	‐	5	
	AV‐5	‐	‐	‐	1	
	AV‐7a	‐	‐	1	‐	
	AV‐8	‐	‐	1	‐	
	AV‐19/64	1	‐	15	60	
	AV‐37	‐	1	5	15	
0.45^1^	HRV‐14	0.5	5	‐	15	12062394
0.5^3^	DHBV	‐	0.5	‐	2	17011665
	AV‐5	5	‐	‐	60	15142717
	AV‐26	‐	0.5	2	5	
	AV‐44	0.5	‐	‐	60	
0.5^1^	PV‐1	‐	10	‐	60	19482374
	ECHO‐1	‐	5	‐	10	
0.5	AV‐5	‐	025	0.5	1	9403252
	HSV‐1	‐	‐	0.25	0.5	
	RV	‐	‐	0.5	5	
	MV	‐	‐	‐	0.5	
	IVA	‐	‐	‐	0.25	
	ROV	0.25	0.5	5	10	
	PV‐1	0.5	‐	5	‐	
	CV‐B3	0.5	‐	10	15	
	PV‐3	0.5	1	5	15	
0.8^4^	MVA	‐	‐	‐	0.25	26381737
	EBOV	‐	‐	‐	0.25	
0.8^6^	FCV	‐	‐	‐	1	9949965
0.9^1^	HSV‐1	‐	‐	‐	0.5	10754445
	CV‐A9	0.5‐15	‐	‐	‐	
	AV‐8	1	2	‐	5	
0.9	IAV	‐	‐	‐	0.25	27009506
	PV‐1	‐	15	‐	30	
	AV‐3	‐	1	‐	5	
1.0^5^	MNV	‐	‐	‐	0.25	22293670
1.0^7^	MNV	‐	‐	‐	0.5	18378650
1.0	AV‐5	‐	0.25	0.5	1	9403252
	HSV‐1	‐	‐	0.25	0.5	
	MAV	‐	‐	‐	0.5	
	IVA	‐	‐	‐	0.25	
	ROV	‐	0.25	1	10	
	PV‐1	0.5	5	10	‐	
	HRV	‐	0.25	‐	‐	
	HIV	‐	‐	0.5	‐	
	IAV	‐	‐	‐	10 s	16490988
1.8^1^	AV‐8	1	2	‐	5	10754445
2.0	ROV	‐	0.25	0.5	5	9403252
	PV‐1	0.5	5	10	‐	
	CV‐B3	0.5	‐	‐	‐	
	PV‐3	0.5	3		‐	
	AV‐3	‐	1	5	15	30589605
	AV‐4	‐	‐	‐	1	
	AV‐5	‐	‐	‐	1	
	AV‐7a	‐	‐	1	‐	
	AV‐8	‐	1	5	‐	
	AV‐19/64	1	5	‐	15	
	AV‐37	1	5	15	60	
2.5^3^	AV‐5	15	‐	‐	‐	15142717
	AV‐26	‐	0.5	2	15	
	AV‐44	0.5	60	‐	‐	
3.2^8^	MERS‐CoV	‐	‐	‐	0.25	26416214
	MVA	‐	‐	‐	0.25	
4.5^1^	HSV‐1	‐	‐	‐	0.5	10754445
	CV‐A9	‐	5‐15	‐	‐	
	AV‐8	1	2	‐	5	
4.5^9^	FCV	‐	‐	‐	10 s	22451431
	CV‐A7	‐	‐	‐	10 s	
	CV‐B5	‐	‐	10 s	1	
	AV‐3	‐	10 s	1	‐	
	AV‐7	‐	10 s	1	‐	
	AV‐8	10 s	1	‐	‐	
5.0^10^	PV‐1	‐	‐	‐	15	6099370
5.0	AV‐3	‐	‐	1	15	30589605
	AV‐4	‐	‐	1	60	
	AV‐5	‐	‐	‐	1	
	AV‐7a	‐	‐	1	‐	
	AV‐8	‐	1	5	‐	
	AV‐19/64	1	15	60	‐	
	AV‐37	‐	1	‐	60	
	AV‐5	‐	‐	0.25	1	9403252
	RV	0.5	1	‐	‐	
	MV	‐	‐	‐	0.5	
	IVA	‐	‐	‐	0.25	
	ROV	‐	‐	0.25	0.5	
	HRV	‐	0.25	‐	‐	
	HIV	‐	‐	0.5	‐	
6.0^8^	MERS‐CoV	‐	‐	‐	0.25	26416214
	MVA	‐	‐	‐	0.25	
4.0/8.0^1^	VV	‐	‐	‐	0.5	20536707
	BVDV	‐	‐	‐	0.5	
	PolyV	‐	‐	‐	0.5	
	AV‐5	‐	0.5	1	3	
	PV‐1	15	30	‐	60	
8.0^4^	MVA	‐	‐	‐	0.25	26381737
	EBOV	‐	‐	‐	0.25	
9.0^1^	HCV	1	‐	‐	‐	25527548
9.0	IAV	‐	‐	‐	0.25	27009506
	PV‐1	30	60	‐	‐	
	AV‐3	5	15	30	60	
10.0	HSV‐1	5	10	‐	‐	2880894
	AV‐5	‐	‐	0.25	3	9403252
	MAV	‐	‐	0.5	10	
	IVA	‐	‐	‐	0.25	
	ROV	‐	‐	0.25	0.5	

Note

No data (‐).

Abbreviations: AV‐3, adenovirus type 3; AV‐4, adenovirus type 4; AV‐5, adenovirus type 5; AV‐7, adenovirus type 7; AV‐7a, adenovirus type 7a; AV‐8, adenovirus type 8; AV‐19/64, adenovirus type 19/64; AV‐26, adenovirus type 26; AV‐37, adenovirus type 37; AV‐44, adenovirus type 44; BVDV, bovine viral diarrhea virus; CV‐A7, coxsackievirus A7; CV‐A9, coxsackievirus A9; CV‐B3, coxsackievirus B3; DHBV, duck hepatitis B virus; EBOV, ebolavirus; ECHO‐1, ECHO virus type 1; FCV, feline calicivirus; HCV, hepatitis C virus; HIV, human immunodeficiency virus; HRV‐14, human rhinovirus type 14; HSV‐1, herpes simplex virus type 1; IAV, influenza A virus; MAV, measles virus; MERS‐CoV, Middle East respiratory syndrome coronavirus; MNV, murine norovirus; MV, mumps virus; MVA, modified vaccinia virus Ankara; n.e., not effective; PolyV, polyomavirus; PV‐1, poliovirus type 1; PV‐3, poliovirus type 3; ROV, rotavirus; RV, rubella virus; s, seconds; SARS‐CoV, severe acute respiratory syndrome coronavirus; VV, vaccinia virus.

^1^ Basic product: Betaisodona^®^ (10% PVP‐I, Mundipharma, Limburg, Germany).

^2^ Basic product: Isodine^®^ (7% PVP‐I, Fukuchi Pharmaceutical Co, Ltd., Hinocho Gamou‐Gun, Japan).

^3^ Basic product: liposomal PVP‐I (4.25% PVP‐I, Mundipharma, Limburg, Germany).

^4^ Basic product: Betadine (10% PVP‐I, Mundipharma, Limburg, Germany).

^5^ Basic product: Isodine^®^ solution (10% PVP‐I, Meiji Seika Pharma, Tokyo, Japan).

^6^ Basic product: Sanichick (1.6% PVP‐I, Scott and Holiday, Sydney, Australia).

^7^ Basic product: Betadine dermique (10% PVP‐I, Viatris, Merignac, France).

^8^ Basic product: Betadine (7.5% PVP‐I, Mundipharma, Limburg, Germany).

^9^ Basic product: Isodine Palm (5% PVP‐I, Meiji Seika Pharma, Tokyo, Japan).

^10^ Basic product: Betadine (5% PVP‐I, Sarget, Saint‐Julien, France).
